# Visfatin Destabilizes Atherosclerotic Plaques in Apolipoprotein E–Deficient Mice

**DOI:** 10.1371/journal.pone.0148273

**Published:** 2016-02-05

**Authors:** Bo Li, Yunhe Zhao, Hui Liu, Bin Meng, Jitao Wang, Tianjun Qi, Hui Zhang, Tao Li, Peiqing Zhao, Hui Sun, Jia Xu, Haibo Song, Zhe Dong, Fengshuang An

**Affiliations:** 1 Department of Cardiology, Central Hospital of Zibo, Zibo, PR China; 2 Department of Cardiology, Qilu Hospital of Shandong University, Ji’nan, PR China; 3 Prenatal Diagnosis Center, Zibo Maternal and Child Health Hospital, Zibo, PR China; 4 Department of Cardiology, China-Japan Friendship Hospital, Chaoyang District, Beijing, China; William Harvey Research Institute, Barts and The London School of Medicine and Dentistry, Queen Mary University of London, UNITED KINGDOM

## Abstract

**Objectives:**

Although there is evidence that visfatin is associated with atherogenesis, the effect of visfatin on plaque stability has not yet been explored.

**Methods:**

In vivo, vulnerable plaques were established by carotid collar placement in apolipoprotein E–deficient (ApoE^−/−^) mice, and lentivirus expressing visfatin (lenti-visfatin) was locally infused in the carotid artery. The lipid, macrophage, smooth muscle cell (SMC) and collagen levels were evaluated, and the vulnerability index was calculated. In vitro, RAW264.7 cells were stimulated with visfatin, and the MMPs expressions were assessed by western blot and immunofluorescence. And the mechanism that involved in visfatin-induced MMP-8 production was investigated.

**Results:**

Transfection with lenti-visfatin significantly promoted the expression of visfatin which mainly expressed in macrophages in the plaque. Lenti-visfatin transfection significantly promoted the accumulation of lipids and macrophages, modulated the phenotypes of smooth muscle cells and decreased the collagen levels in the plaques, which significantly decreased the plaque stability. Simultaneously, transfection with lenti-visfatin significantly up-regulated the expression of MMP-8 in vivo, as well as MMP-1, MMP-2 and MMP-9. Recombinant visfatin dose- and time-dependently up-regulated the in vitro expression of MMP-8 in macrophages. Visfatin promoted the translocation of NF-κB, and inhibition of NF-κB significantly reduced visfatin-induced MMP-8 production.

**Conclusions:**

Visfatin increased MMP-8 expression, promoted collagen degradation and increased the plaques vulnerability index.

## Introduction

Atherosclerotic plaque rupture and subsequent thrombotic occlusion is considered the leading cause of acute myocardial infarction and stroke. The rupture-prone atherosclerotic plaques are characterized by large lipid cores, thin fibrous caps, increased macrophage infiltration and diminished collagen synthesis as well as decreased accumulation of smooth muscle cells (SMCs) [1, 2].

In recent years, there has been growing interest in understanding the involvement of adipocytokines in the development of cardiovascular complications. Visfatin, which was firstly found in the visceral fat and is also known as nicotinamide phosphoribosyl-transferase (Nampt) and pre-B-cell-colony-enhancing factor (PBEF), plays an important role in a variety of metabolic and stress responses. Visfatin also exhibits proliferative, anti-apoptotic, pro-inflammatory and pro-angiogenic properties[3]. Visfatin/PBEF/Nampt can be synthesized and mainly released by visceral fat[4], especially perivascular fat of the vessels [5], such as the aorta or coronary artery. In addition, activated monocytes/macrophages are also important sources of visfatin [6]. Visfatin has a positive association with coronary artery disease (CAD) and acute myocardial infarction, and there is strong visfatin immunostaining in plaques [6]. It has been reported that visfatin induced leukocyte adhesion to endothelial cells [7], enhanced the expression of IL-6 [8] and IL-8 [8] and induced a pro-coagulant phenotype in human coronary endothelial cells by promoting tissue factor expression [9]. In addition, visfatin induces the expression and activity of MMP-2 and MMP-9 [10, 11], which are key enzymes that facilitate the fragility of atherosclerotic plaques.

According to the above results, visfatin might have a role in weakening plaque stability. However, on the other hand, visfatin has been reported to promote collagen synthesis in rat cardiac fibroblasts via the p38MAPK, PI3K, and ERK 1/2 pathways [12]. Meanwhile, visfatin stimulates vascular smooth muscle cell (VSMC) proliferation via ERK1/2 and p38 signaling [5]. Both collagen and SMCs, the main components of the fibrous cap, are thought to have irreplaceable roles in preventing plaque rupture.

Therefore, although there are numerous studies on visfatin, the direct and precise effects of visfatin on plaque stability and thrombus formation have not yet been fully defined. In the present study, a series of in vivo and in vitro experiments was designed and performed to investigate the exact role of visfatin on morphological changes in plaque composition that are associated with increased risk of disruption.

## Materials and Methods

### Reagents

A lentiviral vector containing the coding sequence of the visfatin gene was commercially sourced from Invitrogen (Shanghai, China). Recombinant human visfatin was purchased from Sigma-Aldrich (St. Louis, MO). Rabbit polyclonal anti-GAPDH was purchased from Cell Signaling Technology Inc. (Danvers, MA). Rabbit monoclonal anti-visfatin was purchased from Abcam (Cambridge, UK). Rabbit polyclonal antibodies to α-smooth muscle cell actin and MMP-8 were both purchased from Abcam (Cambridge, UK). Rat anti–mouse monoclonal antibody for macrophages was purchased from Abcam (Cambridge, UK). Rabbit polyclonal antibodies to phospho-NF-κB (p65) and phospho-IκBα were both purchased from Cell Signaling Technology Inc. (Danvers, MA). BAY11-7082 and SC-514, selective inhibitors of NF-κB, were both purchased from Sigma-Aldrich (St. Louis, MO). Rabbit polyclonal antibodies to smooth muscle-myosin heavy chain (SM-MHC), 22 kDa smooth muscle protein (SM22α), smooth muscle calponin (SM-Calponin), smooth muscle myosin light chain kinase (SM-MLCK), h-Caldesmon (h-CALD), Ki-67 and Osteopontin were all purchased from Bioss (Beijing, China). Rabbit polyclonal antibodies to MMP-1, MMP-2 and MMP-9 were also purchased from Bioss (Beijing, China).

### Preparation of lentivirus

Mouse Visfatin gene sequence was retrieved from GeneBank (NM_021524). Firstly, plenti6.3-MCS-IRES-EGFP vector was digested with restriction endonucleases BamH I and Xhol I. Then, the Visfatin insert and linearized plasmid were joined by T4-DNA ligase and then characterized by polymerase chain reaction (PCR), restriction endonuclease digest and sequencing analysis. Lentiviral Visfatin overexpression vector was then transfected into the 293T packaging cells, and high-level lentivirus particles in culture supernatant were collected after about 48 h culture. After 50000g ultracentrifugation for 2 hours, supernatant of the viral particle stock was removed. At last, the viral was resuspended in opti-MEM cultivation liquid and preserved in—80°C.

### Animal protocol

All animal work was performed in compliance with the Animal Management Rules of the Chinese Ministry of Health (Document No. 55, 2001) and was approved by the Animal Care Committee of Shandong University (Jinan, China). Male apolipoprotein E (ApoE^-/-^) mice (n = 80, 6 weeks of age) were purchased from Peking University (Beijing, China). As previously described [13, 14], after 2 weeks of a chow diet (5% fat and no added cholesterol), mice were given a constrictive silastic tube (0.30 mm inner diameter, 0.50 mm outer diameter, and 2 mm length; Shandong Key Laboratory of Medical Polymer Materials, Jinan, China), which was placed around the left common carotid artery. They were then given a high-fat diet (16% fat and 0.25% cholesterol) for 8 weeks. Eight weeks after the collar placement, the mice were randomly divided into 2 groups, the lenti-null group (n = 40, local injection of 1×10^7^ TU null lentivirus in left carotid artery region) and the lenti-visfatin group (n = 40, local injection of 1×10^7^ TU lentivirus containing visfatin in the left carotid artery region). Local lentiviral infection was performed as previously described [15]. Briefly, mice were anesthetized with pentobarbital sodium at a concentration of 40 mg/kg via intraperitoneal injection, and the silastic tubes were removed. The proximal right common carotid artery and the distal right internal and external carotid arteries were temporarily ligated. Then lentiviral suspensions were instilled into the left common carotid artery and were left *in situ* for 15 min. At last, the ligations were released and the skin wounds were closed with silk sutures. Finally, the mice were given a high-fat diet for another 4 weeks.

### Tissue preparation and histological analysis

As described in detail previously [13, 14], at the end of the experiments, all mice were anesthetized with intraperitoneal injection of pentobarbital, and blood samples were collected. Mice were perfused with physiological saline through the left ventricle and were then perfusion-fixed with 4% paraformaldehyde at a pressure of 100 mmHg for histological and morphological staining. The other mice were only perfused with physiological saline and then quickly stored at -80°C for real-time PCR and Western blot. The left common carotid arteries were collected and serial cryosections (5 μm) were prepared. The lipid content was identified using Oil Red O staining. Collagen was visualized using Sirius red staining. Macrophages and vessel smooth muscle cells were detected by immunohistochemistry. Briefly, sections were rehydrated in PBS (pH 7.4) for 30 min and incubated with 3% H_2_O_2_ for 10 min to inhibit endogenous peroxidase activity. After blocking with 5% bovine serum albumin (BSA) for 30 min, sections were incubated with primary antibodies overnight at 4°C, which was followed by treatment with appropriate secondary antibodies for 2 h at 37°C. Then, a 3,3’-diaminobenzidine staining kit (ZSGB-Bio, Beijing, China) was used and the sections were counterstained with hematoxylin. Corresponding sections were immunostained with the following antibodies: anti-visfatin polyclonal antibody (1:100), anti-monocyte/macrophage monoclonal antibody (MOMA-2, 1:150), anti-α-smooth muscle actin monoclonal antibody (1:100), and anti-matrix metalloproteinase (MMP-8) monoclonal antibody (1:500). Serial sections were cut at 5 μm thickness every 50 μm along the carotid artery specimens. The site with the maximal plaque size was selected for morphologic analysis. The average lesion areas and positive areas were quantified with ImagePro-Plus software (Media Cybernetics)[16].

### Vulnerability index

To precisely calculate the vulnerable index, at least 10 high-power fields (400×) at 50 μm intervals were evaluated, and the component areas of the lipids, macrophages, SMCs and collagen were quantitated. The vulnerable index was calculated with the following formula: the relative positive staining areas of (macrophages % + lipid %) / the relative positive staining areas of (SMCs % + collagen %) [13, 14].

### Cell culture

RAW264.7 cells were obtained from American Type Culture Collection (Manassas, VA, USA) and cultured in DMEM (Gibco) supplemented with 10% FBS, 10 μg/ml streptomycin and 100U/ml penicillin. To investigate the effects of visfatin on MMPs expression, RAW264.7 macrophages were cultured and treated with different concentrations of visfatin (0, 0.5, 5, 50 and 500 ng/ml) or different time points (0, 3, 6, 12, 24 and 48 h). To further ascertain the signal transduction pathway involved, RAW264.7 macrophages were cultured again and treated with different time points (0, 2, 4, 8 and 12 h) of visfatin (500ng/ml).

### Western blot analysis

The tissue and cell proteins were extracted with a radioimmunoprecipitation assay (RIPA) (Beyotime, Jiangsu, China), while the nuclear protein and cytoplasmic proteins of macrophages were extracted with a nuclear protein extraction kit (Beyotime, China) for evaluation of NF-κB expression. The protein concentration was measured using the bicinchoninic acid (BCA) method. Protein lysates were subjected to electrophoresis in 10% sodium dodecyl sulfate-polyacrylamide gel (SDS-PAGE) at 90 V and transferred to nitrocellulose paper (Millipore, USA) at 200 mA for 2 hours. Afterwards, the nitrocellulose paper was blocked with 5% non-fat milk for 1 h at room temperature and then washed three times with TBST (Tris-buffered saline containing 0.2% Tween-20). Membranes were incubated with primary antibodies overnight at 4°C. After three washes with TBST, nitrocellulose paper was probed with horseradish peroxidase (HRP) conjugated secondary Ab for an additional two hours at room temperature. Finally, the antigen-antibody complex was detected with an enhanced chemiluminescence (ECL) detection system (Millipore, USA). The results were analyzed with Quantity One (Bio-Rad, USA)[16, 17]. The dilution ratios of the primary antibodies and corresponding secondary antibodies were as follows: anti-visfatin (1:1000, secondary antibody: 1:10000), MMP-8 (1:200, secondary antibody: 1:15000), MMP-1 (1:200, secondary antibody: 1:15000), MMP-2 (1:100, secondary antibody: 1:15000), MMP-9 (1:100, secondary antibody: 1:15000), and GAPDH (1:2000, secondary antibody: 1:10000).

### Quantitative real-time PCR

Total RNA was extracted from frozen common carotid artery specimens with TranZol Up (Trans, China). The RNA quality and quantity were analyzed by spectrophotometry. A 1 μg sample of mRNA was reverse transcribed using the First Strand cDNA Synthesis Kit (Fermentas, UAB). The amplification of the total cDNA was performed with the Light Cycler (Roche, Basel, Switzerland) real-time PCR detection system using the First Start Universal SYBR Green Master (ROX) (Roche, Swiss Confederation) for 40 cycles at 95°C for 10 sec, 60°C for 20 sec, and 72°C for 30 sec. PCR was performed in duplicate, with 18S rDNA chosen as the reference gene. The primer sequences for real-time PCR analyses were as follows: mouse visfatin, forward primer: 5’- GGCCACAAATTCCAGAGAACAG-3’ and reverse primer: 5’-CCAAATGAGCAGATGCCCCTAT-3’;[18] mouse MMP-8, forward primer: 5’- GTAAACTGTAGAGTCGATGC-3’ and reverse primer: 5’-CATAGGGTGCGTGCAAGGAC-3’;[19] and 18S, forward primer: 5’-CTTAGTTGGTGGAGCGATTTG-3’ and reverse primer: 5’- GCTGAACGCCACTTGTCC-3’ [17].

### Immunofluorescence analysis

Immunofluorescence analysis was performed as previously described [17, 20]. Briefly, for tissue sections, after incubation with H_2_O_2_ and BSA, the cryosections were simultaneously incubated with primary antibodies against MOMA-2 (diluted 1:150) and visfatin (diluted 1:100) to colocalize macrophages and visfatin. Rhodamine-conjugated goat anti-rat IgG (ZSGB-Bio, Beijing, China and diluted 1:100) and FITC-conjugated goat anti-rabbit IgG (ZSGB-Bio, diluted 1:100) were used as secondary antibodies. RAW264.7 cells, cultured in 24-well plates, were treated with recombinant human visfatin. After stimulation, cells were fixed with 4% paraformaldehyde for 10 min and blocked with goat serum for 30 min. Then, cells were incubated with a primary antibody against MMP-8 (diluted 1:200), and FITC-conjugated goat anti–rabbit IgG was used as a secondary antibody. Then, immunolabeled cells were counterstained with DAPI (ZSGB-Bio) and sealed with antifade reagent. Positive immunostained areas were also analyzed with Image-Pro Plus 5.0.

### Statistical analysis

Predictive analytics software (PASW) Statistics 18.0 (SPSS Inc., Chicago, IL) was used to analyze the data. All data were expressed as the mean ± SEM. The normally distributed data were analyzed by one-way ANOVA, and the nonparametric variables were analyzed with the Mann-Whitney U test. *P*<0.05 was considered statistically significant.

## Results

### Up-regulation of Visfatin expression in atherosclerotic plaques

**Fig 1:**
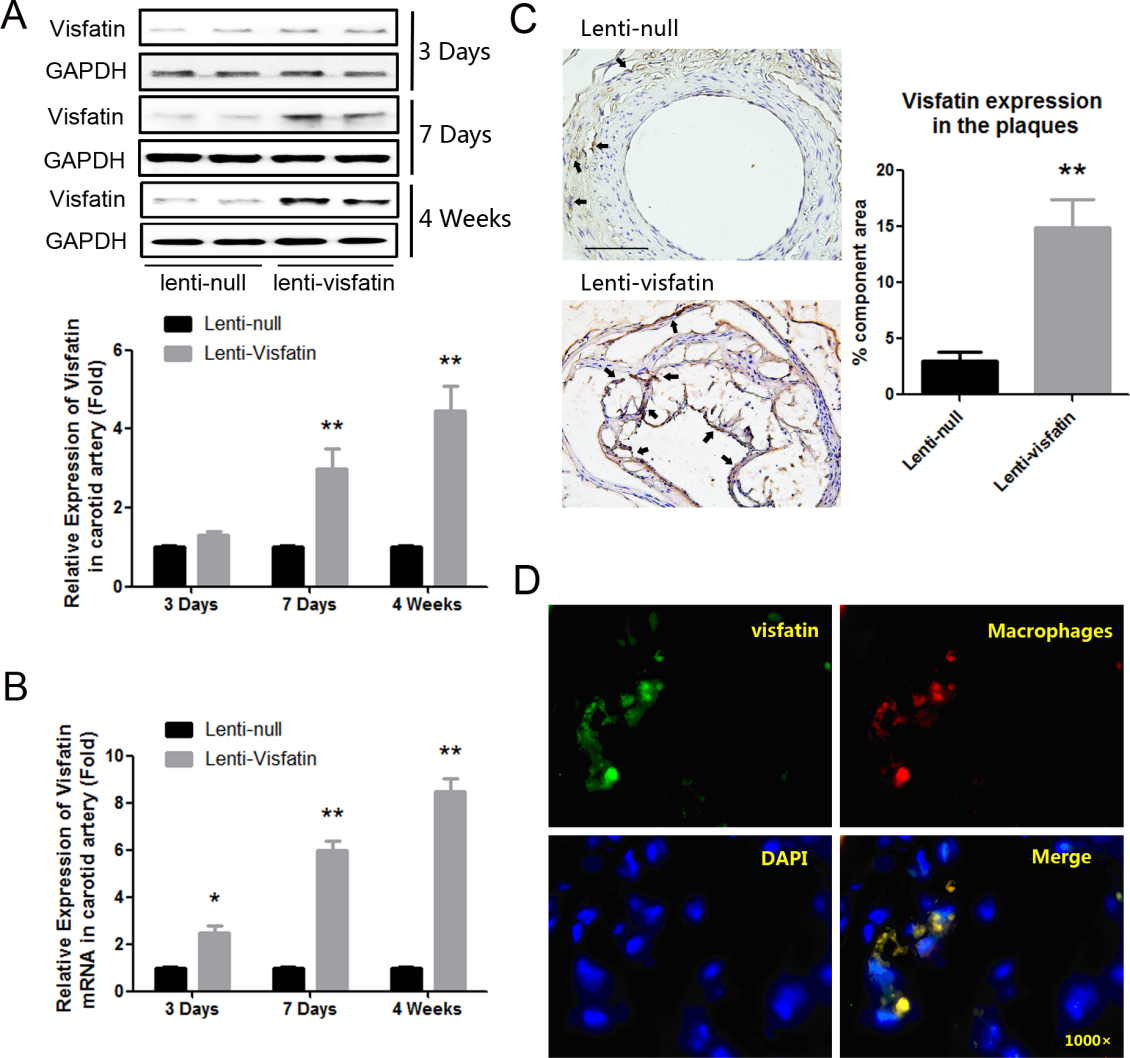
Expression of Visfatin in the lenti-null group and lenti-visfatin group. A, Western blot analysis was used to detect the visfatin expression in the represent samples of carotid plaques from the lenti-null group and lenti-visfatin group respectively after lentivirus transduction for 3 days, 7 days and 4 weeks. B, Real-time PCR was used to detect the visfatin mRNA expression in the carotid plaques of the lenti-null group and lenti-visfatin group. C, Immunochemical stainings of visfatin in the plaques in the lenti-null group and lenti-visfatin group after lentivirus transduction for 4 weeks were shown. The positive staining areas are indicated in brown. D, The co-localization of visfatin (green) with macrophages (red), according to immunofluorescence staining, is shown. Scale bar: 100μm. Arrows are indicative of representative visfatin staining positively regions. ** P<0.01 versus the lenti-null group. Data shown are the means ± SEM.

To determine the effects of visfatin on the stability of atherosclerotic plaques, visfatin lentivirus was used to locally infiltrate the atherosclerotic carotid arteries. In order to further determine the transduction efficiency, we respectively extracted the protein and mRNA from the carotids of the mice after lentivirus transduction for 3 days, 7 days and 4 weeks, and detected the expression of visfatin at both protein and mRNA levels. The results demonstrated that mRNA level (P<0.05, Fig 1B), but not protein level (P>0.05, Fig 1A), was significantly enhanced after 3 days post-transduction. However, both mRNA level and protein level were significantly enhanced after 7 days post-transduction (P<0.01, Fig 1A and 1B). After 4 weeks, the transfection efficiency of visfatin in the carotid plaques was detected again, and the expression of visfatin was still significantly up-regulated in the lenti-visfatin group compared with the lenti-null group (P<0.01, Fig 1A, 1B and 1C). The results above demonstrated that transfection of visfatin lentivirus successfully up-regulated the expression of visfatin in the murine carotid arteries. As described in previous studies, macrophages have been thought to be the main source of visfatin in the plaques [6]. In the present study, to further confirm where visfatin is generated in the plaques, we colocalized visfatin and macrophages in carotid lesions. As shown in Fig 1D, the immunofluorescence confirmed that although other cells which cannot be labeled by MOMA-2 antibody could also express small amount of visfatin, the majority of visfatin originated from macrophages.

### Roles of Visfatin on the carotid plaque composition and stability

**Fig 2:**
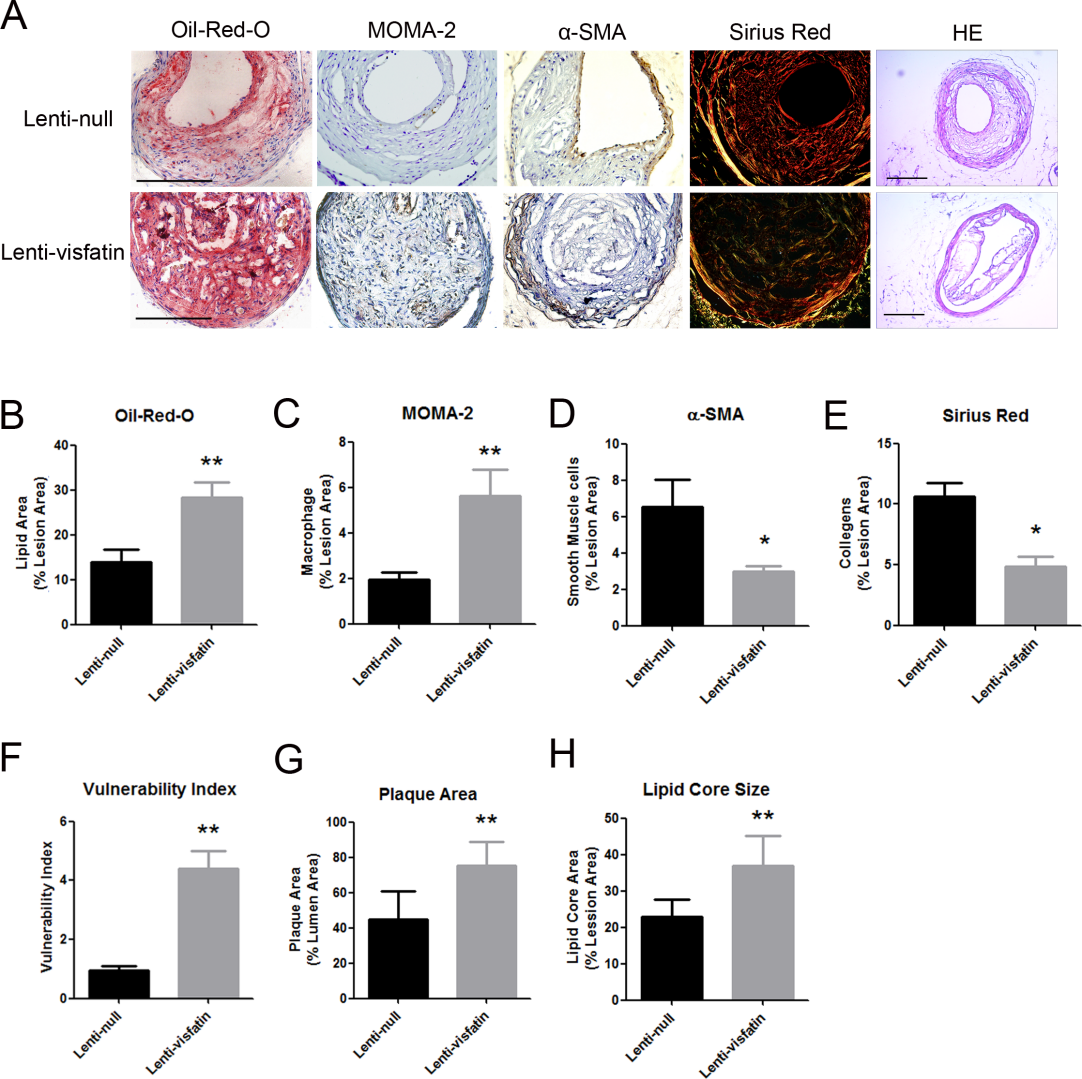
Effects of lenti-visfatin transfection on the plaque composition. A, Staining for the carotid plaques lipids (red), macrophages (brown), SMCs (brown), collagens (red, yellow and green) and HE staining in 2 treatment groups is shown. B, Quantitative analysis of the lipids (n = 20 in each group). C, Quantitative analysis of the monocytes/macrophages (n = 20 in each group). D, Quantitative analysis of the VSMCs (n = 20 in each group). E, Quantitative analysis of the collagen (n = 20 in each group). F, The vulnerability indexes were analyzed in 2 treatment groups. Scale bars: 100μm. G, Quantitative analysis of plaque size in lumens (n = 20 in each group). H, Quantitative analysis of lipid core size in lumens (n = 20 in each group). *P<0.05 versus the lenti-null group, **P<0.01 versus the lenti-null group. Data shown are the means ± SEM.

To investigate the precise effect of visfatin on plaque stability, the levels of lipids, macrophages, smooth muscle cells and collagen in plaques were detected (Fig 2A), and the vulnerable indexes were also calculated. The ratio of the lipids areas to the total lesion areas of the lenti-visfatin transfection group was significantly elevated compared with the lenti-null group (*P*<0.01, Fig 2B). The relative area of macrophages in the lenti-visfatin group was also significantly elevated compared with the lenti-null group (*P*<0.01, Fig 2C). However, the relative area of SMCs in the lenti-visfatin group was also significantly decreased compared with the lenti-null group (*P*<0.05, Fig 2D). The relative collagen levels in the lenti-visfatin group were also greatly diminished compared with the lenti-null group (*P*<0.05, Fig 2E). Consequently, the vulnerability index of the lenti-visfatin group was much higher than that of the lenti-null control group (*P*<0.01, Fig 2F). Meanwhile, the results of HE staining were also shown (Fig 2A). The plaque size of lenti-visfatin group was significantly increased compared with the lenti-null group (*P*<0.01, Fig 2G) and thus leading to the degree of luminal stenosis increased. After that, we also calculated the sizes of the lipid cores of the 2 groups, and the results demonstrated that the lipid core size was also larger in lenti-visfatin group than that in the lenti-null group (*P*<0.01, Fig 2H). These results demonstrated that lentivirus transfection of visfatin changed the composition of carotid plaques and significantly increased the plaque fragility.

### Roles of Visfatin on the body weight and serum profiles

**Fig 3:**
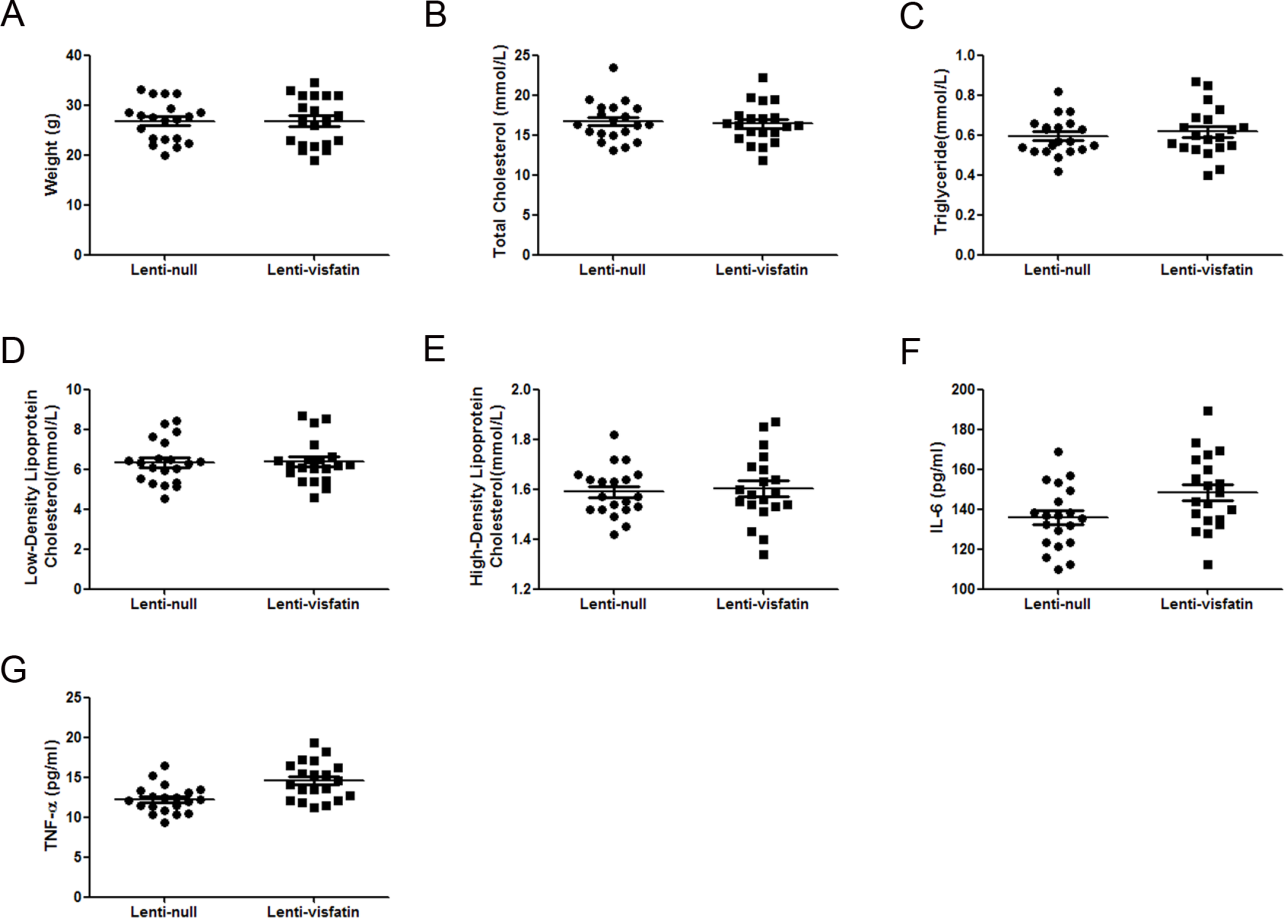
Effects of visfatin on the body weight and serum profiles. A, Lenti-visfatin did not have a significant effect on the body weight compared with the lenti-null group (P>0.05). B-E, Lenti-visfatin did not have a significant effect on the serum lipid profiles compared with the lenti-null group (P>0.05). F-G, Lenti-visfatin did not have a significant effect on the IL-6 and TNF-α inflammation markers compared to the lenti-null group (P>0.05). Data shown are the means ± SEM.

During the rearing period, no local or systemic adverse effects were found in the 2 groups of ApoE^-/-^ mice. We found no significant difference in the body weight of the lenti-visfatin group compared with the lenti-null group (*P*>0.05, Fig 3A). These results demonstrated that the transfection of lenti-visfatin was safe in these animals. There was still no significant difference in the serum TC, TG, LDL-C and HDL-C levels among the 2 groups of ApoE^-/-^ mice (*P*>0.05, Fig 3B–3E), suggesting that the local transfection of lenti-visfatin did not affect the circulating lipid panel and that the effects of lenti-visfatin transfection were independent of the serum lipid profiles. Compared with lenti-null group, the serum IL-6 and TNF-α levels were slightly increased, but with no statistical difference (*P*>0.05, Fig 3F and 3G), which suggested that the effect of local transfection of lenti-visfatin has no relevance with the circulating serum inflammatory level.

### Role of Visfatin on the expression of MMP-8 in vivo and in vitro

**Fig 4:**
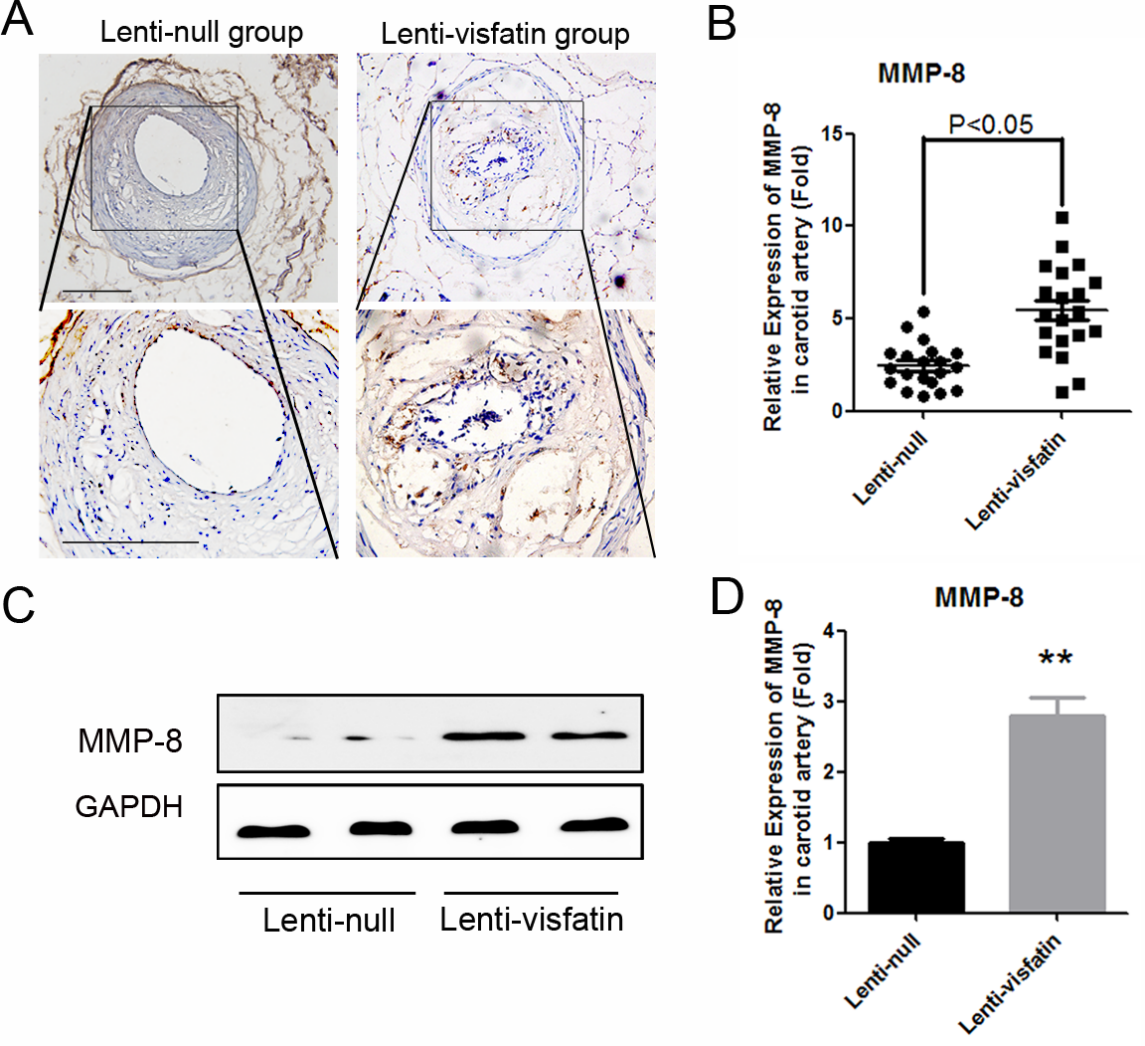
Effects of visfatin on the expression of MMP-8 in vivo. A, Immunochemical staining of MMP-8 in the plaques in 2 treatment groups is shown. The positive staining areas are shown in brown. B, Quantitative analysis of the results in A (n = 20 in each group). C, Western blot analysis was used to detect the expressions of MMP-8 in the represent samples of carotid plaques from the 2 treatment groups. D, Quantitative analysis of the results in C. Scale bars: 100μm. P<0.05 versus the control group.

MMP-8 is one of the most important collagenases that can efficiently degrade collagen. To investigate the mechanism by which lenti-visfatin decreased the expression of collagen in the plaques and destroyed plaque stability, MMP-8 in the carotid plaques was assessed by immunohistochemistry. As shown in Fig 4A–4D, both immunohistochemisty and western blot detections were performed and the results demonstrated that the expression of MMP-8 was significantly enhanced in the lenti-visfatin group compared with the lenti-null group (*P*<0.05).

**Fig 5:**
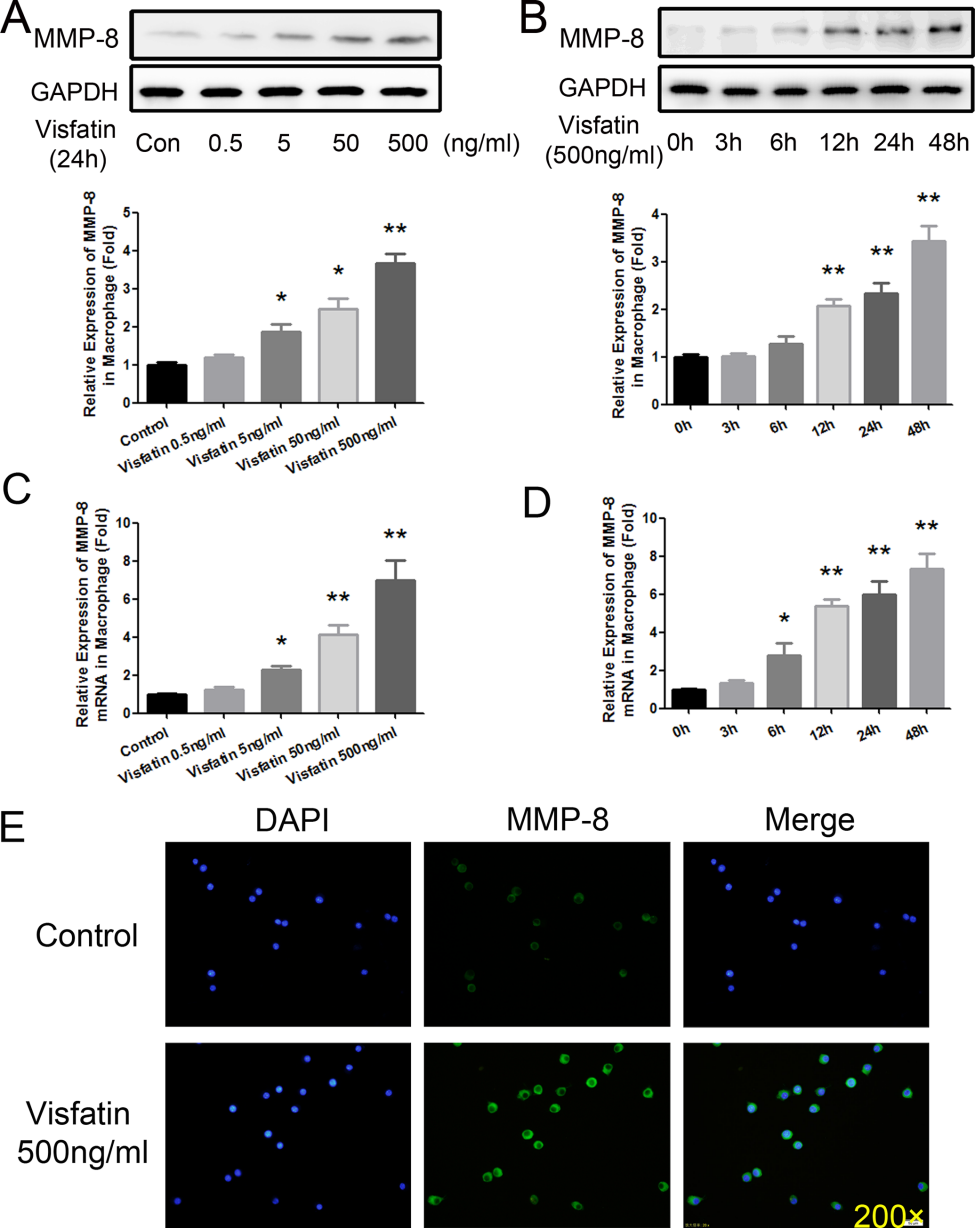
Effects of visfatin on the expression of MMP-8 in vitro. A, Western blot analysis was used to detect the expression of MMP-8 at the protein level after RAW264.7 cells were stimulated for 24 h with different concentration gradients of visfatin (0, 0.5, 5, 50, and 500 ng/ml). B, Western blot analysis was used to detect the expression of MMP-8 at the protein level after RAW264.7 cells were stimulated with 500 ng/ml visfatin for different times (0, 3, 6, 12, 24 and 48 h). C, Real-time PCR was used to detect the expression of MMP-8 at the mRNA level after RAW264.7 cells were stimulated for 24 h with different concentration gradients of visfatin (0, 0.5, 5, 50, and 500 ng/ml). D, Real-time PCR was used to detect the mRNA expression of MMP-8 after RAW264.7 cells were stimulated with 500 ng/ml visfatin for different times (0, 3, 6, 12, 24 and 48 h). E, RAW264.7 cells were incubated with 500 ng/ml visfatin for 24 h. Afterwards, the cells were used for immunofluorescence staining to determine the role of visfatin on MMP-8 (green) expression (magnification 200×). *P<0.05 versus the control group; ** P<0.01 versus the control group. Data shown are the means ± SEM from three independent experiments that were performed in duplicate.

To further ascertain the effect of visfatin on MMP-8 expression, RAW264.7 macrophages were cultured and treated with gradient concentrations of visfatin (0, 0.5, 5, 50 and 500 ng/ml). After 24 h of visfatin treatment, MMP-8 was detected with Western blot, and MMP-8 was up-regulated in a concentration-dependent manner. As shown in Fig 5A, 5 ng/ml of visfatin could significantly elevate the expression of MMP-8 (P<0.05), and the peak elevation was achieved at a concentration of 500 ng/ml (P<0.01). Then, RAW264.7 macrophages were cultured and treated with visfatin at a concentration of 500 ng/ml for different times (0, 3, 6, 12, 24 and 48 h). As shown in Fig 5B, 12 h of stimulation with visfatin could significantly elevate the expression of MMP-8 (P<0.01), which peaked at 48 h (P<0.01). Meanwhile, the results of real-time PCR showed a similar tendency (Fig 5C and 5D). Subsequently, the MMP-8 expression was evaluated using immunofluorescence. After stimulation with visfatin (500ng/ml) for 24 h, RAW264.7 macrophages were examined using immunofluorescence, and there was a significantly elevated effect of visfatin on MMP-8 (Fig 5E). All of these data suggested that visfatin could dramatically improve the expression of MMP-8 in vivo and in vitro, promoting collagen degradation in the arteries and altering the plaque vulnerability index.

### Effect of Visfatin on the expression of MMP-1, MMP-2 and MMP-9

**Fig 6:**
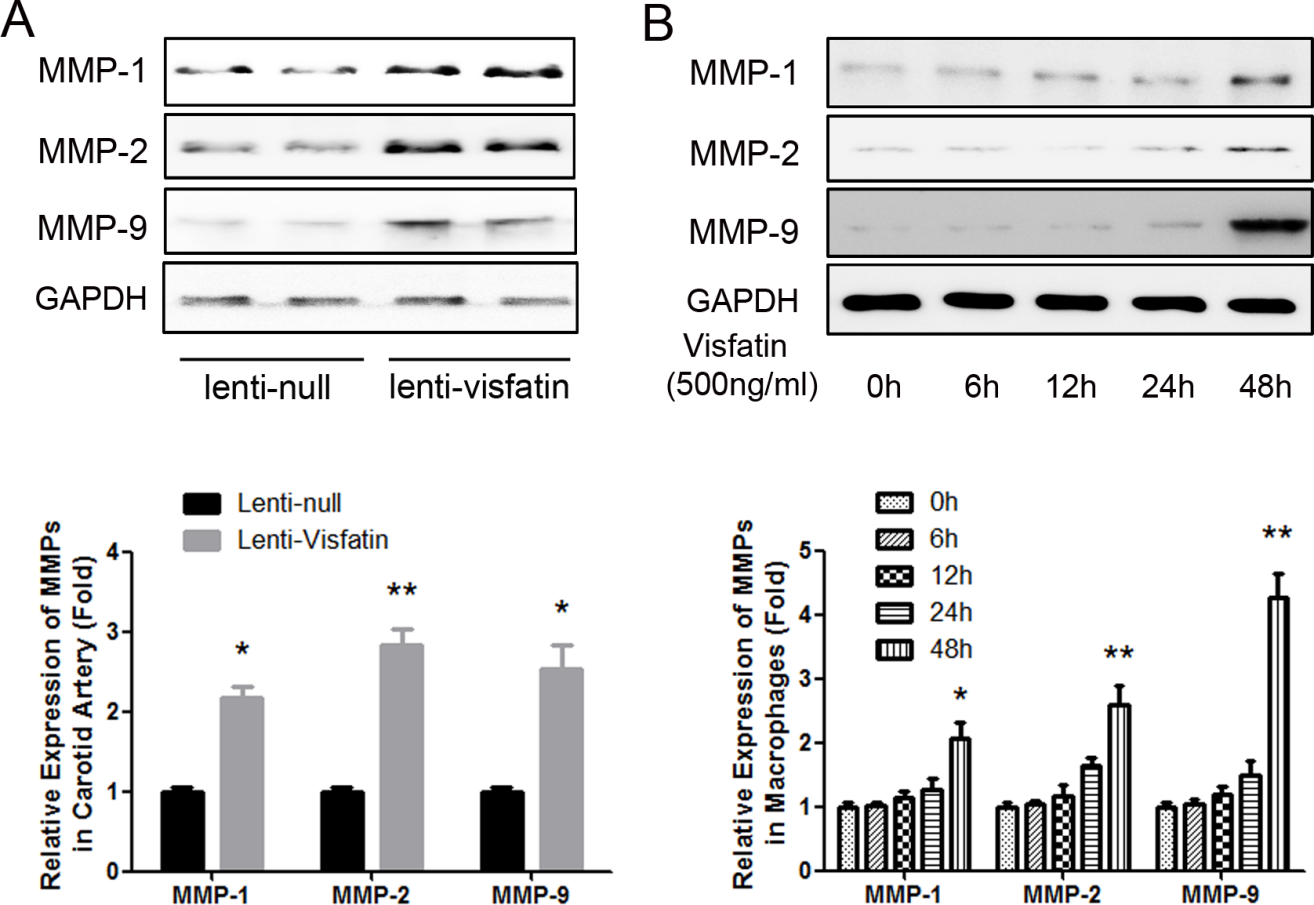
Effect of Visfatin on the Expression of MMP-1, MMP-2 and MMP-9. A, Western blot analysis was used to detect the expressions of MMP-1, MMP-2 and MMP-9 in the represent samples of carotid plaques from the 2 treatment groups. B, RAW264.7 macrophages were cultured and treated with visfatin (500ng/ml) for different time, and western blot analysis was used to detect the expressions of MMP-1, MMP-2 and MMP-9 at different time points. *P<0.05 versus the control group; ** P<0.01 versus the control group. Data shown are the means ± SEM.

In the previous studies, the expressions and activities of MMP-1, MMP-2 and MMP-9 were also reported to be induced by visfatin [10, 11, 21]. Therefore, MMP-1, MMP-2 and MMP-9 in the carotid plaques were assessed with western blot. As shown in Fig 6A, the expressions of MMP-1, MMP-2 and MMP-9 were all significantly increased in the lenti-visfatin group compared with the lenti-null group (*P*<0.05). Then, RAW264.7 macrophages were cultured and treated with visfatin (500ng/ml) for different time, and the results demonstrated that visfatin could not increase the expressions of MMP-1, MMP-2 and MMP-9 in vitro within 24h (Fig 6B). But when we prolonged the stimulation time to 48h, the expressions of MMP-1, MMP-2 and MMP-9 were significantly increased (Fig 6B). Compared with the effect on MMP-8, which was significantly increased after 12h-stimulation, the results of other MMPs reminded us that visfatin might induce MMP-1, MMP-2 and MMP-9 in an indirect way.

### Visfatin Induced MMP-8 Expression via the NF-κB pathway

**Fig 7:**
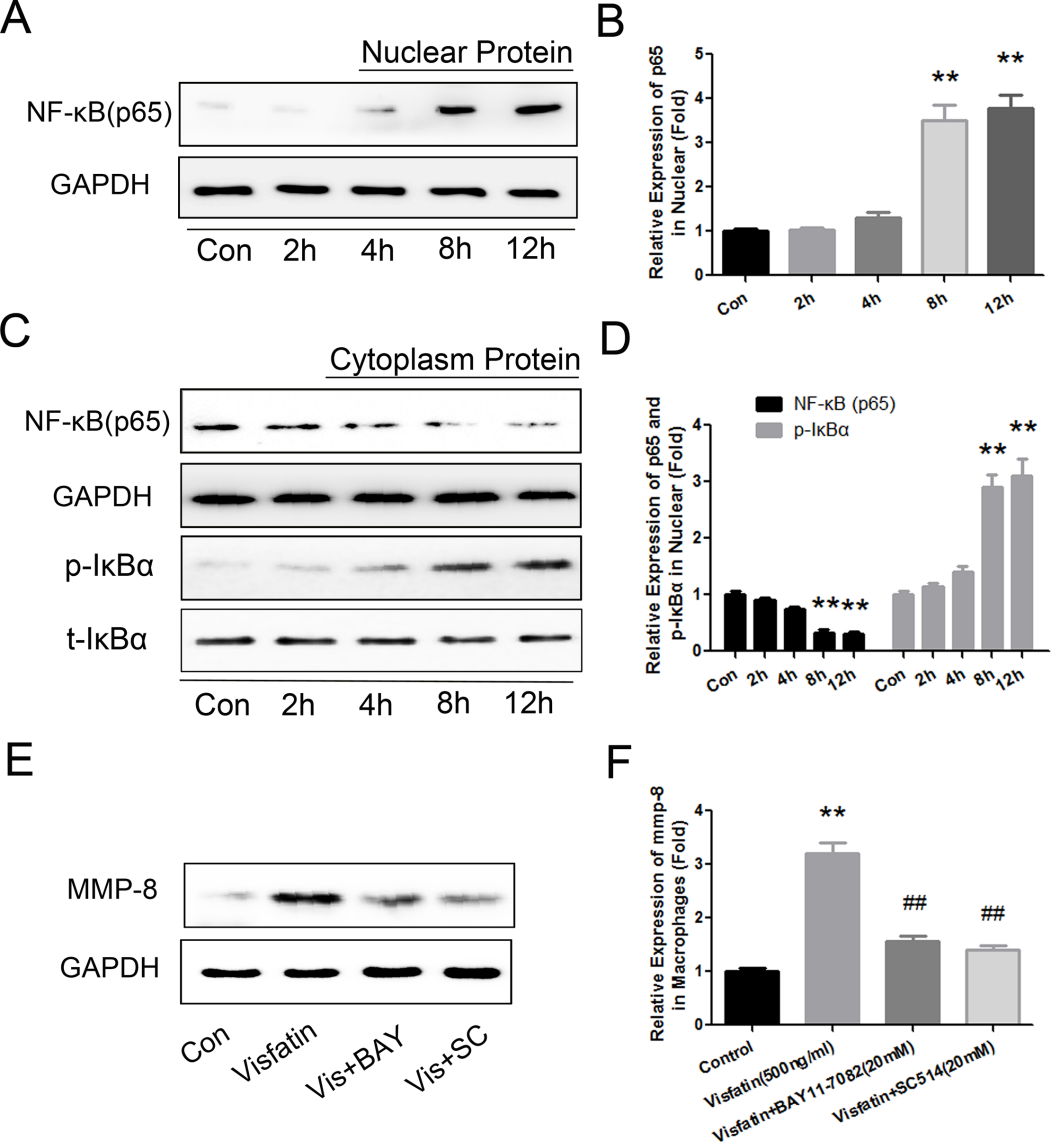
NF-κB mediated visfatin-induced MMP-8 production. RAW264.7 cells were stimulated with 500 ng/ml visfatin for different times (0, 2, 4, 8 and 12 h). Protein was extracted, respectively, from the cytosol and nucleus and the expression levels of p65 and IκBα were, respectively, detected. A and B, Western blot analysis was used to detect the expression of p65 in the nucleus. C and D, Western blot analysis was used to detect the expression of p65 and IκBα in the cytosol. E and F, The NF-κB pathway was blocked with 2 h of treatment with the following inhibitors: BAY11-7082 (20 μM) and SC-514 (20 μM) before visfatin was added to the cells. Western blot analysis was used to detect the expression of MMP-8. ** P<0.01 versus the control group, ## P<0.01 versus the visfatin group. Data shown are the means ± SEM from three independent experiments that were performed in duplicate.

Transcription factor NF-κB was reported to play an important role in the expression and release of MMP-8 in a previous investigation [22]. Simultaneously, NF-κB is one of the most important pathways through which visfatin exerts its biological effects [7, 23]. To determine whether the NF-κB pathway mediated visfatin-induced MMP-8 expression, we extracted protein from the cytosol and nucleus and evaluated the expression of P65 and IκBα. Visfatin (500ng/ml) treatment of RAW264.7 macrophages significantly increased the expression of p65 in the nucleus and decreased its expression in the cytosol in a time-dependent manner (Fig 7A–7D). Meanwhile, the treatment also increased the phosphorylation of IκBα in the cytosol (Fig 7C and 7D). Both of the effects on p65 and IκBα reached statistical significance after 8 h treatment, and they peaked after 12 h of treatment. Then, to further determine the effect of the NF-κB pathway on the reaction, we blocked the NF-κB pathway with 2 h of treatment with two inhibitors, BAY11-7082 (20 μM) and SC-514 (20 μM), before visfatin was added to the cells. Both of the inhibitors significantly suppressed visfatin-induced MMP-8 production (Fig 7E and 7F). All of the results indicated that the activation and translocation of NF-κB is critical for visfatin-induced MMP-8 production.

## Discussion

The major findings of the present study are as follows: (1) Visfatin induced the changes of the structure and composition of the plaques, and increased the vulnerability of the plaques; (2) Visfatin affects plaque MMP-8, which might reflect the mechanism underlying the observed change in plaque vulnerability index. (3) Visfatin induced the expression of MMP-8 via NF-κB pathway.

It is well known that vulnerable plaques possess the following characteristics: increased lipid core size, increased macrophage infiltration, decreased numbers of smooth muscle cells and decreased collagen content [1, 2]. Among the novel adipocytokines reported in recent years, visfatin is one of the most important, and it has been frequently investigated for its association with atherosclerotic diseases and the related cells around atherosclerotic plaques [6]. Visfatin has been identified as a non-traditional biomarker of inflammation [24] and atherosclerosis [25]. There is evidence of a positive association between the circulating visfatin level and endothelial dysfunction [24]; simultaneously, the normalization of endothelial function following renal transplantation is accompanied by a reduction in the circulating visfatin levels [26]. Accumulating evidence indicates that visfatin could activate the inflammation status of different cell types, including endothelial cells, VSMCs, and monocytes/macrophages. Visfatin activates the inflammation-related transcription factor, NF-κB [11], which is mediated by the expression of many cytokines in the plaques. Visfatin increases the expression of ICAM-1 and VCAM-1 through ROS-dependent NF-κB activation in endothelial cells [7], which have been thought to be the key molecules involved in macrophage recruitment and the formation of atherosclerotic plaques. Furthermore, visfatin induces tissue factor expression and promotes the transition of the human coronary endothelial cells to a pro-coagulant phenotype [9]. Visfatin was also reported to induce the expressions and activities of MMP-2 and MMP-9 [10, 11], as well as MMP-1 [21], and therefore contribute to the degradation of collagen and the extracellular matrix (ECM), resulting in atherosclerotic plaque rupture.

Nevertheless, although visfatin possesses the aforementioned properties and might accordingly exert its pro-disrupture effects on atherosclerotic plaques, there is still a lack of direct evidence of its role in atherosclerotic plaques. Conversely, visfatin has been reported to promote collagen synthesis [12] and stimulate VSMC proliferation [5]; both of these are the main components of the fibrous cap and can prevent plaque rupture. Because of the contradictory pieces of evidence, we cannot easily describe the precise role of visfatin on the stability of atherosclerotic plaques.

In the present study, to elucidate the precise effect of visfatin on plaque stability, lentivirus with high expression of visfatin was synthesized, amplified, and injected into the carotid artery. Oil Red O staining showed that lenti-visfatin infiltration could enhance lipid accumulation in the plaques. However, the lentivirus infiltration did not affect the levels of circulating lipids. The results suggested that local transfection of lenti-visfatin did not affect the systemic conditions; instead, it locally played an important role in the carotid plaques. Immunohistochemistry staining showed that lenti-visfatin infiltration could significantly increase the aggregation of monocytes/macrophages, while simultaneously diminish the expression of contractile phenotype markers of VSMCs. As shown in S1 Fig, in order to ascertain the role of visfatin on VSMC, rat VSMC were separated and cultured. After stimulation with visfatin, the contractile phenotype markers (α-SMA, SM-MHC, SM22α, SM-Calponin, SM-MLCK and H-Caldesmon) were decreased, while the proliferative phenotype markers (osteopontin and Ki-67) were increased, which indicated that visfatin has the character to transform contractile phenotype into proliferative phenotype. Then we detected the expression of osteopontin in the plaques, and the result confirmed again that the expression of proliferative phenotype marker of SMC was promoted. In the present study, the results demonstrated that visfatin changed the phenotype of smooth muscle cell. Masson staining also indicated that visfatin inhibits collagen expression. All of the results, as well as the calculation of the vulnerability index, strongly suggested that transfection of lenti-visfatin promoted atherosclerotic plaque vulnerability.

Subsequently, we needed to explore the potential mechanisms of collagen degradation and plaque disruption. Activated MMP-8 can cleave a wide range of extracellular matrix proteins, especially collagen I, which is the most important component of the fibrous cap and which can protect the integrity of the atherosclerotic vessel wall [27]. Shu Ye and his colleagues have found that knocking-out MMP8 gene in ApoE^-/-^ mice resulted in a substantial reduction of atherosclerotic lesion, and had significantly fewer macrophages but increased collagen content in the plaques [28]. A relationship among MMP8 gene variation with atherosclerosis progression was also observed in a population-based study [28]. Afterwards, they further uncovered that MMP-8 played an important role in plaque angiogenesis [29], which was also seen as one of the mechanisms of plaque vulnerability [30]. Dollery and colleagues confirmed that MMP-8, secreted from macrophages is mainly localized in the lesion’s shoulder, a region that is prone to rupture [31]. Moreover, MMP-8 can activate other MMPs and inactivate TIMP-1 [32, 33]. MMP-8 has been considered a leading enzyme that modulates plaque rupture [27, 34]. MMP-8, also known as collagenase-2 or neutrophil collagenase, degrades collagens with 3-fold more potency than MMP-1 and MMP-13 [27, 35, 36]. Also, in our previous study, we found that MMP-8 plays a crucial role in plaque stability in the same animal model as in the present study. In our study, transfection of lenti-visfatin in vivo could significantly increase the expression of MMP-8 in the plaques. Then, to further evaluate the role of visfatin on MMP-8, RAW264.7 macrophages were cultured and stimulated with visfatin. As shown in Fig 5A to 5D, MMP-8 was significantly, time- and concentration-dependently, up-regulated by visfatin at the protein and mRNA levels. Afterwards, macrophages were stimulated with 500 ng/ml visfatin for 24 h, and the immunofluorescence results demonstrated the effect of visfatin on MMP-8.

Because other MMPs have been reported to be increased with the stimulation of visfatin [10, 11], we also detected the expressions of MMP-1, MMP-2 and MMP-9 in the plaques of carotid arteries of the mice which have been transfected with lenti-null or lenti-visfatin. We found that transfection with lenti-visfatin could also increase the expressions of MMP-1, MMP-2 and MMP-9. We then stimulated RAW264.7 macrophages with visfatin in vitro, and the results demonstrated that visfatin could not increase the expressions of MMP-1, MMP-2 and MMP-9 in vitro within 24h. But when we prolonged the stimulation time to 48h, the expressions of MMP-1, MMP-2 and MMP-9 were significantly increased. Compared with the effect on MMP-8, which was significantly increased after 12h-stimulation, the results of other MMPs reminded us that visfatin might induce MMP-1, MMP-2 and MMP-9 in an indirect way. Therefore, according to the results above, we thought that MMP-8 played a more important role than the other MMPs.

Furthermore, we found that visfatin could significantly induce both the translocation of NF-κB (p65) from the cytosol to the nucleus, which can mediate multiple biological reactions, and the synthesis and release of many cytokines, including MMP-8[22]. Activation and translocation of NF-κB by most agents requires phosphorylation of its inhibitory subunit, IκBα, which could mask the nuclear localization signals (NLS) of NF-κB proteins and therefore keep NF-κB sequestered in an inactive state in the cytoplasm[37]. We also found that visfatin promoted the phosphorylation of IκBα in the cytoplasm as well as further inhibited the translocation of NF-κB. The application of NF-κB pathway inhibitors further confirmed that visfatin induced MMP-8 production via the NF-κB pathway.

Despite of the new findings, there are still some limitations in the present study. For instance, we showed the method to establish an atherosclerotic animal model involved in plaque stability, which could reflect the changes of the structure and composition of the carotid arteries and provided us a practical tool for the investigation of atherosclerotic plaque stability. But the originations and the developments of this kind of lesions resulted from artificial local vessel stenosis, which might different from the plaques formed under natural state. In addition, transfection the lentivirus containing visfatin gene to mice also gave us a higher expression level, which help us know about the changes of the arteries led by the specifically and locally over-expression and achieve the purpose of the experiments. However, after all, the over-expression was non-physiological, and no one could accurately estimate what the virus transfected into the mice would bring in. All in all, according to the results of the present study, regulation of the local visfatin expression level might be a new approach for protecting atherosclerotic plaques. Also, further studies are needed to validate the conclusion and clarify the underlying molecular signaling mechanisms.

## Supporting Information

S1 Fig Visfatin decreased the expression of contractile phenotype markers, but increased the expression of proliferative phenotype markers. A, Immunofluorescence staining showed that visfatin dramatically decreased the expression of contractile phenotype markers, α-SMA and SM-MHC, in cultured rat smooth muscle cells. B, Western blot analysis showed that visfatin dramatically decreased the expression of contractile phenotype markers: SM22α, SM-Calponin, SM-MLCK and H-Caldesmon, but increased the expression of proliferative phenotype markers: osteopontin and Ki-67. C, Immunochemical staining of osteopontin in the plaques in 2 groups is shown. The positive staining areas are shown in brown. D, Quantitative analysis of the results of osteopontin in 2 groups (n = 20 in each group). Scale bar: 100μm. P<0.01 versus the control group.
(TIF)

## References

[1] Falk E, Shah PK, Fuster V. Coronary plaque disruption. Circulation 1995, 92(3):657–671. 7634481 10.1161/01.cir.92.3.657

[2] Finn AV, Nakano M, Narula J, Kolodgie FD, Virmani R. Concept of vulnerable/unstable plaque. Arteriosclerosis, thrombosis, and vascular biology 2010, 30(7):1282–1292. 10.1161/ATVBAHA.108.179739 20554950

[3] Dalamaga M. Nicotinamide phosphoribosyl-transferase/visfatin: a missing link between overweight/obesity and postmenopausal breast cancer? Potential preventive and therapeutic perspectives and challenges. Medical hypotheses 2012, 79(5):617–621. 10.1016/j.mehy.2012.07.036 22922056

[4] Sandeep S, Velmurugan K, Deepa R, Mohan V. Serum visfatin in relation to visceral fat, obesity, and type 2 diabetes mellitus in Asian Indians. Metabolism: clinical and experimental 2007, 56(4):565–570. 17379018 10.1016/j.metabol.2006.12.005

[5] Wang P, Xu TY, Guan YF, Su DF, Fan GR, Miao CY. Perivascular adipose tissue-derived visfatin is a vascular smooth muscle cell growth factor: role of nicotinamide mononucleotide. Cardiovascular research 2009, 81(2):370–380. 10.1093/cvr/cvn288 18952695

[6] Dahl TB, Yndestad A, Skjelland M, Oie E, Dahl A, Michelsen A, et al Increased expression of visfatin in macrophages of human unstable carotid and coronary atherosclerosis: possible role in inflammation and plaque destabilization. Circulation 2007, 115(8):972–980. 17283255 10.1161/CIRCULATIONAHA.106.665893

[7] Kim SR, Bae YH, Bae SK, Choi KS, Yoon KH, Koo TH, et al Visfatin enhances ICAM-1 and VCAM-1 expression through ROS-dependent NF-kappaB activation in endothelial cells. Biochimica et biophysica acta 2008, 1783(5):886–895. 10.1016/j.bbamcr.2008.01.004 18241674

[8] Lee WJ, Wu CS, Lin H, Lee IT, Wu CM, Tseng JJ, et al Visfatin-induced expression of inflammatory mediators in human endothelial cells through the NF-kappaB pathway. Int J Obes (Lond) 2009, 33(4):465–472. 19223849 10.1038/ijo.2009.24

[9] Cirillo P, Di Palma V, Maresca F, Pacifico F, Ziviello F, Bevilacqua M, et al The adipokine visfatin induces tissue factor expression in human coronary artery endothelial cells: another piece in the adipokines puzzle. Thrombosis research 2012, 130(3):403–408. 10.1016/j.thromres.2012.06.007 22726553

[10] Adya R, Tan BK, Punn A, Chen J, Randeva HS. Visfatin induces human endothelial VEGF and MMP-2/9 production via MAPK and PI3K/Akt signalling pathways: novel insights into visfatin-induced angiogenesis. Cardiovascular research 2008, 78(2):356–365. 18093986 10.1093/cvr/cvm111

[11] Adya R, Tan BK, Chen J, Randeva HS. Nuclear factor-kappaB induction by visfatin in human vascular endothelial cells: its role in MMP-2/9 production and activation. Diabetes care 2008, 31(4):758–760. 10.2337/dc07-1544 18184904

[12] Yu XY, Qiao SB, Guan HS, Liu SW, Meng XM. Effects of visfatin on proliferation and collagen synthesis in rat cardiac fibroblasts. Horm Metab Res 2010, 42(7):507–513. 10.1055/s-0030-1249059 20225169

[13] Li JJ, Meng X, Si HP, Zhang C, Lv HX, Zhao YX, et al Hepcidin destabilizes atherosclerotic plaque via overactivating macrophages after erythrophagocytosis. Arterioscler Thromb Vasc Biol 2012, 32(5):1158–1166. 10.1161/ATVBAHA.112.246108 22383698

[14] Li B, Luo BB, Qin WD, Liu H, Xia YF, Liu TX, et al Bidirectional effect of serum amyloid A on plaque stability. International journal of cardiology 2014, 174(1):179–183. 10.1016/j.ijcard.2014.03.186 24742813

[15] Yang JM, Wang Y, Qi LH, Gao F, Ding SF, Ni M, et al Combinatorial interference of toll-like receptor 2 and 4 synergistically stabilizes atherosclerotic plaque in apolipoprotein E-knockout mice. Journal of cellular and molecular medicine 2011, 15(3):602–611. 10.1111/j.1582-4934.2010.01028.x 20132416 PMC3922382

[16] Zhang XC, Chen JQ, Li B. Salvianolic acid A Suppresses CCL-20 Expression in TNF-alpha -Treated Macrophages and ApoE Deficient Mice. Journal of cardiovascular pharmacology 2014. 10.1097/FJC.0000000000000117 24853487

[17] Li B, Dong Z, Liu H, Xia YF, Liu XM, Luo BB, et al Serum amyloid A stimulates lipoprotein-associated phospholipase A2 expression in vitro and in vivo. Atherosclerosis 2013, 228(2):370–379. 10.1016/j.atherosclerosis.2013.03.023 23623642

[18] Segawa K, Fukuhara A, Hosogai N, Morita K, Okuno Y, Tanaka M, et al Visfatin in adipocytes is upregulated by hypoxia through HIF1alpha-dependent mechanism. Biochemical and biophysical research communications 2006, 349(3):875–882. 16970912 10.1016/j.bbrc.2006.07.083

[19] Xu L, Peng H, Wu D, Hu K, Goldring MB, Olsen BR, et al Activation of the discoidin domain receptor 2 induces expression of matrix metalloproteinase 13 associated with osteoarthritis in mice. J Biol Chem 2005, 280(1):548–555. 15509586 10.1074/jbc.M411036200

[20] Zhang XC, Chen JQ, Li B. Salvianolic acid A suppresses CCL-20 expression in TNF-alpha-treated macrophages and ApoE-deficient mice. Journal of cardiovascular pharmacology 2014, 64(4):318–325. 10.1097/FJC.0000000000000117 24853487

[21] Nokhbehsaim M, Eick S, Nogueira AV, Hoffmann P, Herms S, Frohlich H, et al Stimulation of MMP-1 and CCL2 by NAMPT in PDL cells. Mediators Inflamm 2013, 2013:437123 10.1155/2013/437123 24058270 PMC3766615

[22] La VD, Tanabe S, Bergeron C, Gafner S, Grenier D. Modulation of matrix metalloproteinase and cytokine production by licorice isolates licoricidin and licorisoflavan A: potential therapeutic approach for periodontitis. J Periodontol 2011, 82(1):122–128. 10.1902/jop.2010.100342 20722535

[23] Fan Y, Meng S, Wang Y, Cao J, Wang C. Visfatin/PBEF/Nampt induces EMMPRIN and MMP-9 production in macrophages via the NAMPT-MAPK (p38, ERK1/2)-NF-kappaB signaling pathway. Int J Mol Med 2011, 27(4):607–615. 10.3892/ijmm.2011.621 21327328

[24] Bessa SS, Hamdy SM, El-Sheikh RG. Serum visfatin as a non-traditional biomarker of endothelial dysfunction in chronic kidney disease: an Egyptian study. European journal of internal medicine 2010, 21(6):530–535. 10.1016/j.ejim.2010.09.011 21111939

[25] Kadoglou NP, Sailer N, Moumtzouoglou A, Kapelouzou A, Tsanikidis H, Vitta I, et al Visfatin (nampt) and ghrelin as novel markers of carotid atherosclerosis in patients with type 2 diabetes. Experimental and clinical endocrinology & diabetes: official journal, German Society of Endocrinology [and] German Diabetes Association 2010, 118(2):75–80. 10.1055/s-0029-1237360 19834878

[26] Yilmaz MI, Saglam M, Carrero JJ, Qureshi AR, Caglar K, Eyileten T, et al Normalization of endothelial dysfunction following renal transplantation is accompanied by a reduction of circulating visfatin/NAMPT. A novel marker of endothelial damage? Clinical transplantation 2009, 23(2):241–248. 19402217 10.1111/j.1399-0012.2008.00921.x

[27] Lenglet S, Mach F, Montecucco F. Role of matrix metalloproteinase-8 in atherosclerosis. Mediators of inflammation 2013, 2013:659282 10.1155/2013/659282 23365489 PMC3556866

[28] Laxton RC, Hu Y, Duchene J, Zhang F, Zhang Z, Leung KY, et al A role of matrix metalloproteinase-8 in atherosclerosis. Circulation research 2009, 105(9):921–929. 10.1161/CIRCRESAHA.109.200279 19745165 PMC2853782

[29] Fang C, Wen G, Zhang L, Lin L, Moore A, Wu S, et al An important role of matrix metalloproteinase-8 in angiogenesis in vitro and in vivo. Cardiovascular research 2013, 99(1):146–155. 10.1093/cvr/cvt060 23512982

[30] Moulton KS, Vakili K, Zurakowski D, Soliman M, Butterfield C, Sylvin E, et al Inhibition of plaque neovascularization reduces macrophage accumulation and progression of advanced atherosclerosis. Proceedings of the National Academy of Sciences of the United States of America 2003, 100(8):4736–4741. 12682294 10.1073/pnas.0730843100 PMC153625

[31] Dollery CM, Owen CA, Sukhova GK, Krettek A, Shapiro SD, Libby P. Neutrophil elastase in human atherosclerotic plaques: production by macrophages. Circulation 2003, 107(22):2829–2836. 12771009 10.1161/01.CIR.0000072792.65250.4A

[32] Okada Y, Nakanishi I. Activation of matrix metalloproteinase 3 (stromelysin) and matrix metalloproteinase 2 ('gelatinase') by human neutrophil elastase and cathepsin G. FEBS Lett 1989, 249(2):353–356. 2544455 10.1016/0014-5793(89)80657-x

[33] Itoh Y, Nagase H. Preferential inactivation of tissue inhibitor of metalloproteinases-1 that is bound to the precursor of matrix metalloproteinase 9 (progelatinase B) by human neutrophil elastase. J Biol Chem 1995, 270(28):16518–16521. 7622455 10.1074/jbc.270.28.16518

[34] Molloy KJ, Thompson MM, Jones JL, Schwalbe EC, Bell PR, Naylor AR, et al Unstable carotid plaques exhibit raised matrix metalloproteinase-8 activity. Circulation 2004, 110(3):337–343. 15226217 10.1161/01.CIR.0000135588.65188.14

[35] Hasty KA, Jeffrey JJ, Hibbs MS, Welgus HG. The collagen substrate specificity of human neutrophil collagenase. J Biol Chem 1987, 262(21):10048–10052. 3038863

[36] Knauper V, Murphy G, Tschesche H. Activation of human neutrophil procollagenase by stromelysin 2. Eur J Biochem 1996, 235(1–2):187–191. 8631328 10.1111/j.1432-1033.1996.00187.x

[37] Jacobs MD, Harrison SC. Structure of an IkappaBalpha/NF-kappaB complex. Cell 1998, 95(6):749–758. 9865693 10.1016/s0092-8674(00)81698-0

