# Supplementary figures and images for: Visfatin Destabilizes Atherosclerotic Plaques in Apolipoprotein E–Deficient Mice
Source: PLoS One. 2016 Feb 5;11(2):e0148273. doi: 10.1371/journal.pone.0148273 (PMC4743838; doi:10.1371/journal.pone.0148273)

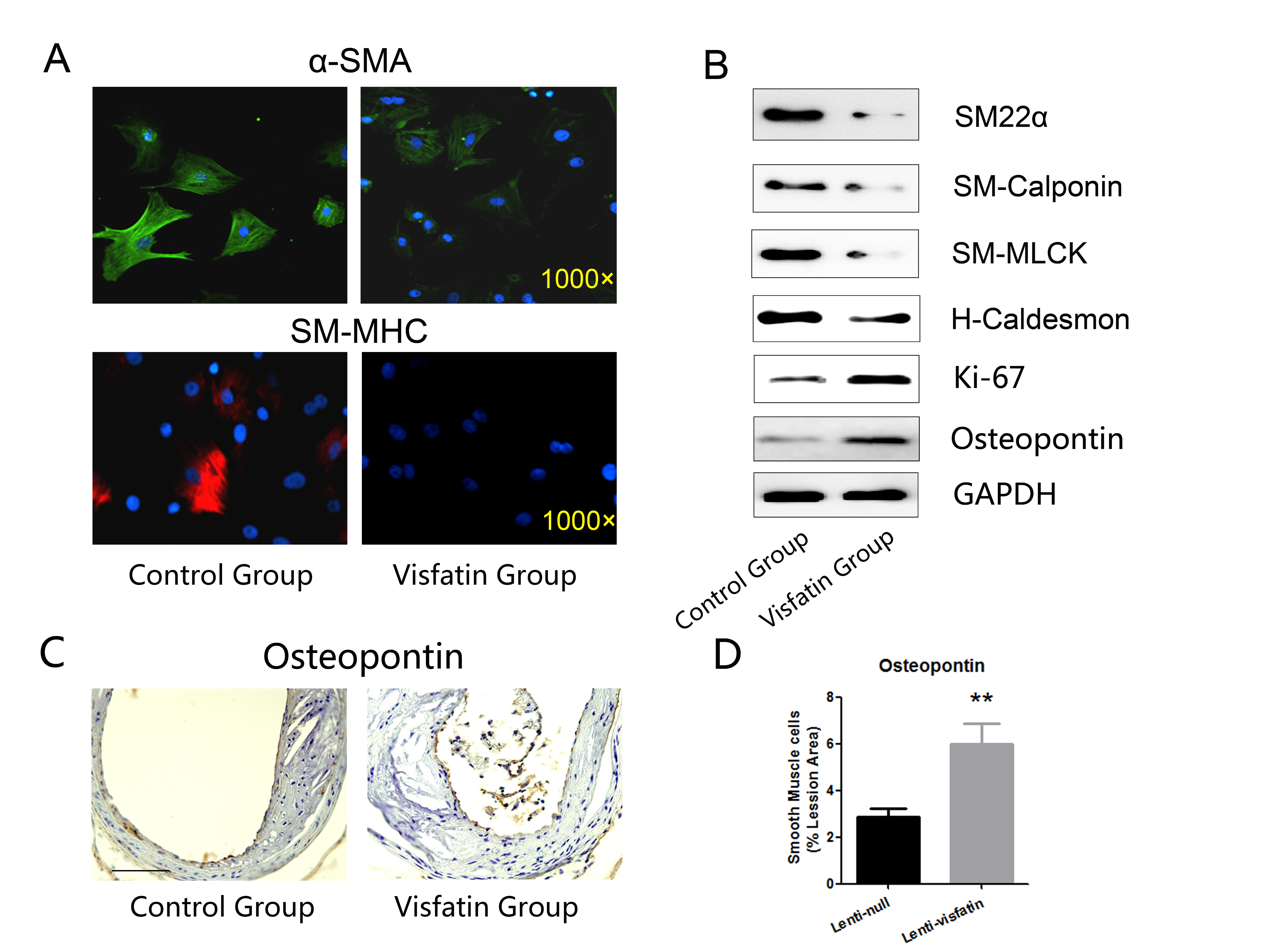

Supplement: S1 Fig — A, Immunofluorescence staining showed that visfatin dramatically decreased the expression of contractile phenotype markers, α-SMA and SM-MHC, in cultured rat smooth muscle cells. B, Western blot analysis showed that visfatin dramatically decreased the expression of contractile phenotype markers: SM22α, SM-Calponin, SM-MLCK and H-Caldesmon, but increased the expression of proliferative phenotype markers: osteopontin and Ki-67. C, Immunochemical staining of osteopontin in the plaques in 2 groups is shown. The positive staining areas are shown in brown. D, Quantitative analysis of the results of osteopontin in 2 groups (n = 20 in each group). Scale bar: 100μm. P<0.01 versus the control group. (TIF) [file pone.0148273.s001.tif]
